# Radiomics-based differentiation of upper urinary tract urothelial and renal cell carcinoma in preoperative computed tomography datasets

**DOI:** 10.1186/s12880-025-01727-9

**Published:** 2025-05-30

**Authors:** Julian Marcon, Philipp Weinhold, Mona Rzany, Matthias P. Fabritius, Michael Winkelmann, Alexander Buchner, Lennert Eismann, Jan-Friedrich Jokisch, Jozefina Casuscelli, Gerald B. Schulz, Thomas Knösel, Michael Ingrisch, Jens Ricke, Christian G. Stief, Severin Rodler, Philipp M. Kazmierczak

**Affiliations:** 1 Department of Urology, University Hospital, LMU Munich, Marchioninistr. 15, 81377 Munich, Germany; 2 Department of Radiology, University Hospital, LMU Munich, Munich, Germany; 3 Department of Pathology, University Hospital, LMU Munich, Munich, Germany; 4 Munich Center for Machine Learning (MCML), Munich, Germany; 5 Department of Urology, University Hospital Schleswig-Holstein, Kiel, Germany

**Keywords:** Machine learning, Radiomics, Renal cell carcinoma, Upper tract urothelial carcinoma

## Abstract

**Background:**

To investigate a non-invasive radiomics-based machine learning algorithm to differentiate upper urinary tract urothelial carcinoma (UTUC) from renal cell carcinoma (RCC) prior to surgical intervention.

**Methods:**

Preoperative computed tomography venous-phase datasets from patients that underwent procedures for histopathologically confirmed UTUC or RCC were retrospectively analyzed. Tumor segmentation was performed manually, and radiomic features were extracted according to the *International Image Biomarker Standardization Initiative*. Features were normalized using z-scores, and a predictive model was developed using the *least absolute shrinkage and selection operator* (LASSO). The dataset was split into a training cohort (70%) and a test cohort (30%).

**Results:**

A total of 236 patients [30.5% female, median age 70.5 years (IQR: 59.5–77), median tumor size 5.8 cm (range: 4.1–8.2 cm)] were included. For differentiating UTUC from RCC, the model achieved a sensitivity of 88.4% and specificity of 81% (AUC: 0.93, radiomics score cutoff: 0.467) in the training cohort. In the validation cohort, the sensitivity was 80.6% and specificity 80% (AUC: 0.87, radiomics score cutoff: 0.601). Subgroup analysis of the validation cohort demonstrated robust performance, particularly in distinguishing clear cell RCC from high-grade UTUC (sensitivity: 84%, specificity: 73.1%, AUC: 0.84) and high-grade from low-grade UTUC (sensitivity: 57.7%, specificity: 88.9%, AUC: 0.68). Limitations include the need for independent validation in future randomized controlled trials (RCTs).

**Conclusions:**

Machine learning-based radiomics models can reliably differentiate between RCC and UTUC in preoperative CT imaging. With a suggested performance benefit compared to conventional imaging, this technology might be added to the current preoperative diagnostic workflow.

**Clinical trial number:**

Local ethics committee no. 20–179

**Supplementary Information:**

The online version contains supplementary material available at 10.1186/s12880-025-01727-9.

## Introduction

Upper urinary tract urothelial carcinomas (UTUCs) are tumors that arise in the ureter and pyelocaliceal cavities [1]. Accounting for only 5 to 10% of all urothelial carcinomas, UTUCs are rare tumors, with an estimated incidence in the Western population of two cases per 100,000 individuals per year [2, 3]. Contrast-enhanced computed tomography (CT), particularly with a urographic phase, is the imaging reference standard for preoperative staging of UTUC and a widely used modality for differentiating renal masses [4, 5].

Differentiating UTUC from renal cell carcinoma (RCC) using imaging can be challenging, particularly in cases of locally advanced disease. Accurate preoperative diagnosis is critical, as surgical approaches differ for both entities. Localized RCC is typically managed with partial or radical nephrectomy, whereas high-grade or locally advanced UTUC is treated with open radical nephroureterectomy, including bladder cuff excision [1, 5]. Preoperative histological evaluation through endourological biopsy has limitations. Despite advancements, such as *Fluorescence in situ Hybridization* (FISH) [6], the accuracy of histopathological results can be limited by tumor heterogeneity [7]. Additionally, the risk of tumor seeding in UTUC during these procedures remains a subject to debate [8]. Consequently, there is a significant clinical need for non-invasive methods to differentiate UTUC from RCC preoperatively.

Radiomics is an innovative technique that transforms medical images into high-dimensional datasets by extracting quantitative features, such as shape, texture, size/volume and intensity through specialized algorithms. These features have the potential to capture image characteristics beyond what is visually perceptible, including tumor grade, receptor status and markers predictive of therapy response [9, 10].

The aim of this proof-of-concept study was to explore the potential of radiomics for the preoperative differentiation of UTUC and RCC, validated by histopathology. We hypothesized that a machine learning-based model, leveraging a panel of 59 standardized radiomic features, could enable non-invasive differentiation between UTUC and RCC using preoperative CT datasets.

## Materials and methods

### Study approval

This retrospective study was conducted with the approval of the institutional review board (Local clinical ethics committee of the Ludwig-Maximilians-University of Munich approval no. 20–179). Our research was carried out in accordance with the Declaration of Helsinki of the World Medical Association, and informed consent to participate in the study was obtained from all patients.

### Patients

**Fig. 1:**
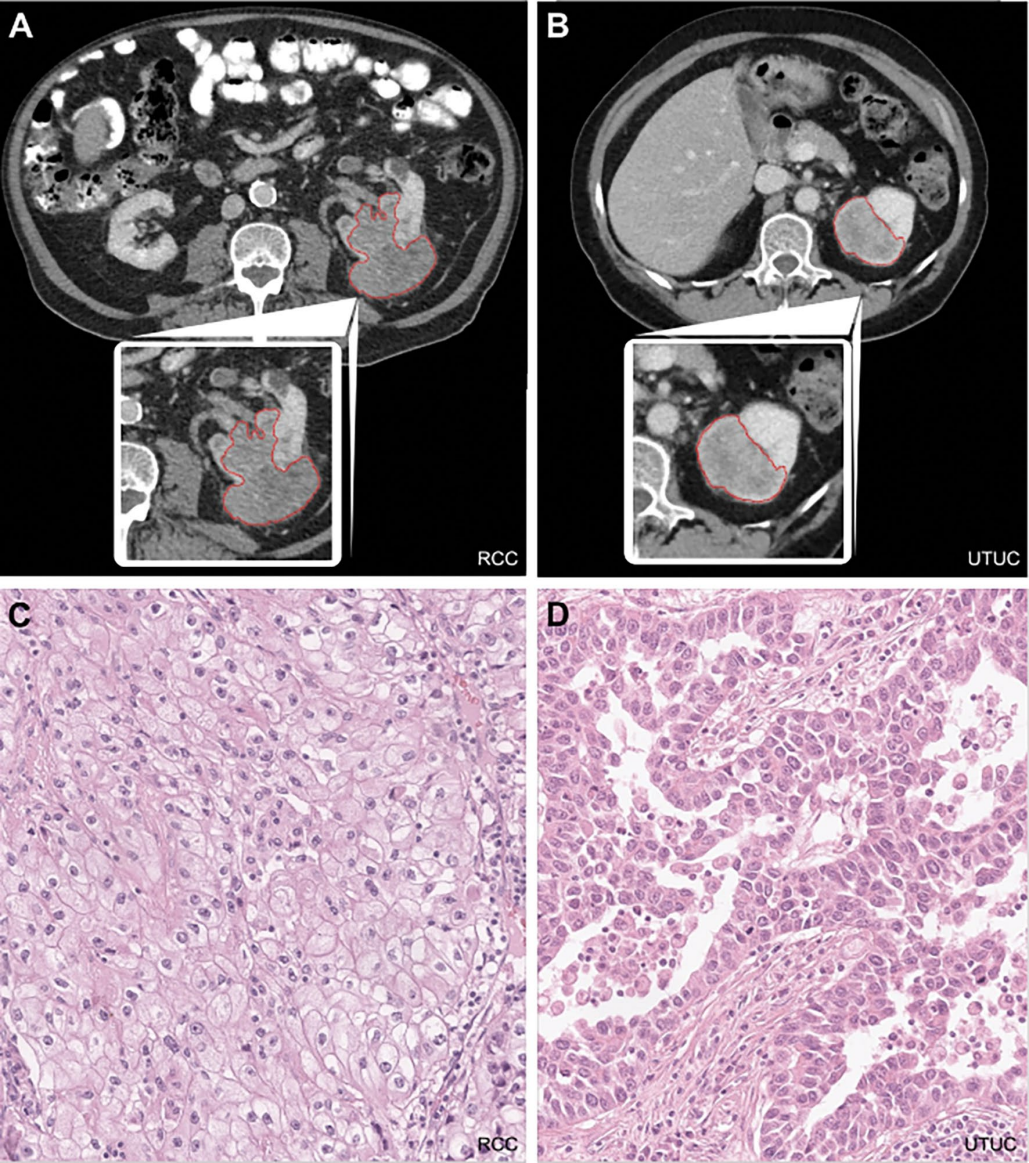
Segmentation of renal tumors on axial series of abdominal CT scans. **A and B** Manual tumor segmentation was performed on CT scans in the venous phase using commercially available software. After detailed examination of the tumor in the various sectional planes, segmentation was performed in the axial plane, starting in the central area of the tumor. In the case of large tumors, each layer was not segmented individually, but the algorithm interpolated individual intermediate layers independently. This was then corrected manually. Tumor segmentations were performed by a trained doctorate student and a board-certified radiologist with more than 10 years of experience in abdominal imaging. **C and D**: Histopathology slides in hematoxylin and eosin staining of ccRCC **(C)** and UTUC **(D)** Abbreviations: RCC: renal cell carcinoma, UTUC: upper tract urothelial carcinoma

Data from patients treated for renal masses at our cross-regional tertiary care academic center between 2005 and 2021 were queried for pretreatment CT imaging. Only patients with pretreatment CT imaging in the venous phase were included to ensure comparability between scans. Patients were eligible if they had a histopathologically confirmed UTUC or RCC, diagnosed via biopsy or resection (exemplary histopathology slides are displayed in Fig. 1C*/D*). Patients with UTUC were included in case of a urothelial carcinoma upon histopathological analysis and if a tumor location in the renal pelvis was identified. UTUCs with ureteral location were excluded. There were no size criteria applied for inclusion of RCC or UTUC tumors. Patients were excluded from the analysis if no pretreatment venous phase CT scan was available and in case of prior treatment of a kidney tumor (surgery, ablative treatment or systemic therapy), tumor recurrence as well as congenital kidney malformations, such as horseshoe kidney or pelvic kidney. Clinical parameters such as age, date of surgery, and histopathological parameters of renal masses (tumor type, tumor subtype, TNM-stage, grading) were collected retrospectively from patient records. The study of this study adhered to the STROBE statement for cohort studies (Suppl. Document 1).

### CT imaging

CT data from our institution and external facilities (secondary care centers, primary care centers, and private practices) were included. All patients received intravenous iodine-based contrast medium. To ensure consistency, only venous-phase scans were analyzed, as this phase is standard in renal tumor CT protocols and provides high lesion-to-parenchyma contrast, even for hypovascular tumors [11]. Soft kernel-reconstructed slice thickness was 3–5 mm, respectively. In addition, although multiphase imaging is considered the reference standard for renal mass characterization, imaging protocols vary substantially across institutions, and corticomedullary or urographic phases are not consistently performed.

### Tumor segmentation and extraction of radiomic features

Tumors were assessed in multiple sectional planes and contrast phases to determine their extent. Manual segmentation of the tumor was performed in the axial plane using commercially available software (mint Lesion™, Mint Medical GmbH, Heidelberg, Germany). Segmentation followed the standards of the International Image Biomarker Standardization Initiative [12].

Radiomic first-order features included standard deviation which reflects the variability of voxel intensities within a lesion and may serve as a proxy for internal heterogeneity, which is often associated with tumor aggressiveness or necrosis. Entropy, as another first-order feature, quantifies the complexity or unpredictability of the intensity distribution and has been linked to tissue disorganization, which may correlate with high-grade or biologically aggressive tumors.

The process began at the tumor center and extended cranially and caudally, ensuring accurate delineation. At the end of the segmentation process, the whole tumor was reviewed and the delineation was corrected, if necessary, until the tumor was accurately segmented. In case of doubt, structures that could not be clearly assigned to the tumor were not included in the volume-of-interest to prevent the extraction of information from extratumoral tissue such as perirenal fat (see Fig. 1A*/B*).

A primary segmentation was performed by a trained specialist and reviewed by a board-certified radiologist with over ten years of experience in abdominal imaging. To assess interreader variability (IRV) and to account for a reliable and reproducible range of radiomic feature values, 30% of datasets were randomly selected for independent review by a second board-certified radiologist with six years of experience. Adjustments were made at the second radiologist’s discretions, and these segmentations were saved separately. Intraclass correlation coefficients (ICC) were calculated to assess interreader agreement.

A total of 59 radiomic features were extracted from the entire delineated tumor volume using the software algorithm. Included features were first order statistics which represent intensities on the voxel level and second order features relating to the spatial distribution of voxels in the grayscale matrix. Gray level co-occurrence matrix (GLCM) features were used as second order texture descriptors to quantify patterns of voxel intensity variations in the ROI by analyzing how frequently pairs of voxel intensities occur at a specific spatial relationship (see Supplementary Table 1). Features achieving an ICC ≥ 0.8 (≥ 80% agreement) were retained for analysis. Additionally, tumor size, represented by long and short tumor axis (measured in mm), was included in the model.

### Machine learning model building and statistical analysis

The cohort was randomly divided into training (70%) and test sets (30%). Radiomic features were normalized using z-scores, to a mean of 0 and a standard deviation of 1. A cross-validated logistic regression model with least absolute shrinkage and selection operator (LASSO) was developed using the *glmnet* engine in R applied for elastic net regression models, to classify samples into RCC or UTUC using radiomics features as predictive variables. The same was done for the classification of a subgroup into ccRCC and high grade UTUC samples, a second subcohort of diagnostically more challenging non-clear cell RCCs (nccRCCs) and UTUCs as well as a third subgroup of high- and low-grade UTUC, based on the different operative management of high-grade disease. Model parameters were tuned for maximum accuracy with 10-fold cross-validation on a grid of penalty values (λ) and the elastic net mixing parameters for lasso regression (α = 1) respectively. The model was trained for λ giving the minimum mean cross-validated error (λ > 0, “lambda.min”). The resulting model was assessed on the remaining testing data; the main evaluation metric was ROC AUC. Model coefficients were inspected to identify predictive features. Non-parametric hypothesis testing was performed to compare baseline characteristics of patients with UTUC and RCC, with a pre-rejection alpha of 0.05. All statistical analyses were performed using R version 4.1.0 (R Core Team 2021).

## Results

A total of 236 patients were included in the study, with 117 (49.6%) diagnosed with UTUC and 119 (50.4%) with RCC. All patients were treated at our university hospital between 2005 and 2021. The demographic and tumor characteristics of the cohort are summarized in Table 1. Female patients comprised 30.5% of the cohort, while 69.5% were male. The median age was 70.5 years (IQR: 59.5–77). The median tumor size for all masses was 5.8 cm (4.1 ± 8.2). The median size of UTUC tumors was 4.2 (2.9 ± 5.8) cm, while RCC tumors were larger, with a median size of 8.1 cm (5.8 ± 9.9).

**Table 1:** Baseline characteristics of the patient cohort

	UTUC (*n* = 117)	RCC (*n* = 119)	*p*-value
**Age**			
Mean [years]	72,1	64,3	**< 0.001**
SD [years]	± 10.8	± 11.5	
**Gender**			
Male	79	85	0.515
Female	38	34	
**Tumor volume**			
Mean [cm³]	37,7	270,6	**< 0.001**
SD [cm³]	62.8	361.0	
**T-Stage**			
Tx	20	1	-
Ta	25	-	
Tis	1	-	
T1	9	27	
T2	6	9	
T3	50	75	
T4	6	7	
**Side of the tumor**			
Left	59	67	0.366
Right	58	52	
**Histology**			-
Clear cell RCC	-	87	
Papillary RCC	-	20	
Chromophobe RCC	-	5	
Sarcomatoid RCC	-	3	
High-grade UTUC	85	-	
Low-grade UTUC	32	-	
Other RCC subtypes	-	4	

**Fig. 2:**
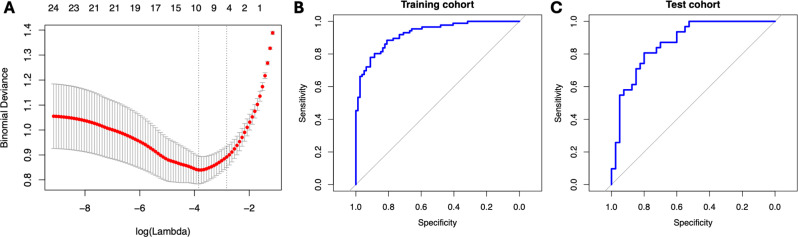
**A** Lasso regularization plot showing the log-transformed values of the regularization parameter, lambda (λ), on the X axis, which controls the penalty applied to the coefficients. The y axis shows the cross-validated error or deviance. The optimal lambda is chosen where the cross-validation error is minimized; **B and C** ROC plots illustrating specificity and sensitivity of the radiomics score established upon Lasso analysis for the distinction between RCC and UTUC

Interreader agreement ≥ 80% was achieved for 28 radiomic features (Supplementary Table 2). These features were included for further analysis. The LASSO regression model identified 10 radiomic features contributing to the radiomic score (see Table 2). Using the score, a differentiation between UTUC and RCC with a sensitivity of 88.4% and a specificity of 81% was observed for the training cohort (AUC: 0.93, Radiomics score cutoff value: 0.467). For the test cohort, the distinction of the two tumor entities was possible with a sensitivity of 80.6% and a specificity of 8 0% (AUC: 0.87, Radiomics score cutoff value: 0.601) (see Fig. 2).

**Table 2:** Predictor values of included radiomic features in the machine learning model

Radiomics feature (raw output)	Feature description [12]	Normalized model coefficient
***Entire cohort (n = 236): UTUC vs. RCC***		
Firstorder.histogram.Entropy	First order feature: Entropy as parameter of randomness in image values	-0.146725622
Firstorder.histogram.Mean	First order feature: Mean gray level intensity in ROI	-0.093077333
Firstorder.histogram.Median.abs.deviation	First order feature: Mean absolute deviation of intensity values from the median value of the image array	-0.108286287
Firstorder.intensity.Robust.mean.abs.deviation	First order feature: Mean absolute deviation of intensity values from the mean value of 10–90% of the image array	-0.384201940
Glcm.Difference.average	Gray level co-occurrence matrix feature: Relationship between pairs of similar and pairs of dissimilar gray level values and	-0.136673047
Glcm.Difference.variance	Gray level co-occurrence matrix feature: Weights means of dissimilar intensity level pairs that differ more from the mean higher	-0.313556241
Glcm.Dissimilarity	Gray level co-occurrence matrix feature: Relationship between pairs of similar and pairs of dissimilar gray level values and	-0.003591719
Glcm.Joint.entropy	Gray level co-occurrence matrix feature: Describes the randomness between neighboring intensity values	0.114638300
Glcm.Std	Gray level co-occurrence matrix feature: Standard deviation of GLCM features	-0.053630813
Tumor.short.axis	Second-largest axis length of the ellipsoid enclosed by the ROI upon segmentation in the CT series	-1.888042670
***Subcohort (n = 172): high grade UTUC vs. ccRCC***		
Firstorder.histogram.Median.abs.deviation	First order feature: Mean absolute deviation of intensity values from the median value of the image array	-0.2014530418
Firstorder.histogram.Std	First order feature: Standard deviation of the distribution of intensity values of the image array (Histogram)	-0.0125926496
Firstorder.Robust.mean.abs.deviation	First order feature: Mean absolute deviation of intensity values from the mean value of 10–90% of the image array	-0.1378609126
Firstorder.intensity.Std	First order feature: Standard deviation of intensity values	-0.0000503306
Glcm.Difference.variance	Gray level co-occurrence matrix feature: Weights means of dissimilar intensity level pairs that differ more from the mean higher	-0.1467854801
Glcm.Std	Gray level co-occurrence matrix feature: Standard deviation of GLCM features	-0.3490017115
Tumor.short.axis	Second-largest axis length of the ellipsoid enclosed by the ROI upon segmentation in the CT series	-1.2137183749
***Subcohort (n = 117): high-grade vs. low-grade UTUC***		
Glcm.Difference.variance	Gray level co-occurrence matrix feature: Weights means of dissimilar intensity level pairs that differ more from the mean higher	-0.2453007
Glcm.Joint.average	Gray level co-occurrence matrix feature: Mean gray level intensity of the analyzed matrix	-0.1000878
Glcm.Std	Gray level co-occurrence matrix feature: Standard deviation of GLCM features	0.0481093
Glcm.Sum.of.averages	Gray level co-occurrence matrix feature: Relationship between pairs of lower intensity and higher intensity values	0.4578403
***Subcohort (n = 144): UTUC vs. nccRCC***		
Firstorder.histogram.Entropy	First order feature: Entropy as parameter of randomness in image values	-0.005620842
Firstorder.intensity.Max	First order feature: Maximum gray level intensity in ROI	-0.209872612
Firstorder.intensity.Root.mean.square	First order feature: Represents the square root of the mean of all squared intensity values and serves as a measure of the overall magnitude of image intensities	0.216805958
Glcm.Joint.entropy	Gray level co-occurrence matrix feature: Describes the randomness between neighboring intensity values	0.063284407
Tumor.short.axis	Second-largest axis length of the ellipsoid enclosed by the ROI upon segmentation in the CT series	-1.676368373

**Fig. 3:**
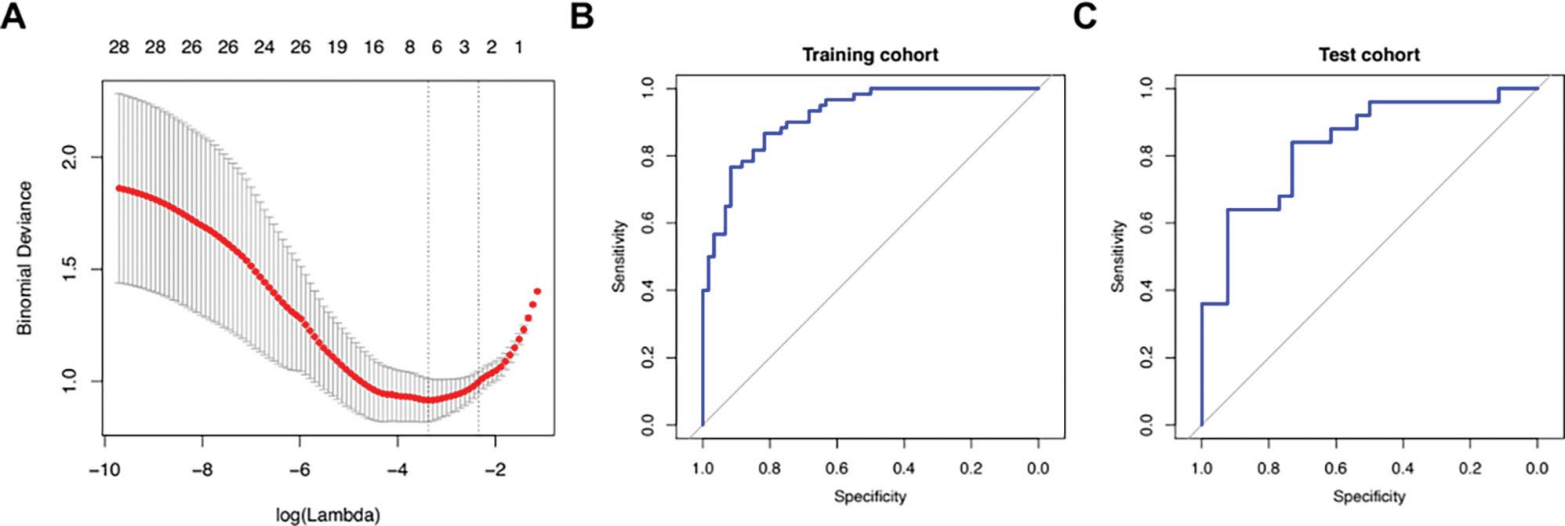
**A** Lasso regularization plot showing the log-transformed values of the regularization parameter, lambda (λ), on the X axis, which controls the penalty applied to the coefficients. The y axis shows the cross-validated error or deviance. The optimal lambda is chosen where the cross-validation error for the outcome differentiation between ccRCC and high grade UTUC is minimized; **B and C** ROC plots illustrating specificity and sensitivity of the radiomics score established upon Lasso analysis for the distinction between ccRCC and high grade UTUC

Testing was then focused on the differentiation between high grade UTUC and ccRCC which comprised 172 cases of our cohort. The radiomic score calculated for this subgroup contained seven features not eliminated by the regression model. In this subgroup a sensitivity of 86.7% and a specificity of 81.7% was obtained for the training cohort regarding the distinction between ccRCC and high grade UTUC (AUC: 0.92, Radiomics score cutoff value: 0.498). For the test cohort, the two variants could be distinguished with a sensitivity of 84% and a specificity of 73.1% (AUC: 0.84, Radiomics score cutoff value: 0.42) (see Fig. 3).

To further evaluate the model’s performance in more diagnostically challenging cases, a subgroup analysis excluding clear cell RCC was performed, comparing non-clear cell RCC (nccRCC) and UTUC (*n* = 144). In the training cohort, the model achieved an AUC of 0.93, with a sensitivity of 69.8%, a specificity of 100%, and an optimal threshold of 0.909. In the independent test cohort, the model demonstrated an AUC of 0.82, with a sensitivity of 91.1%, a specificity of 77.8%, and a threshold of 0.64. These results suggest that the model retains strong discriminative ability even when clear cell RCC, which typically exhibits distinct imaging characteristics, is excluded.

**Fig. 4:**
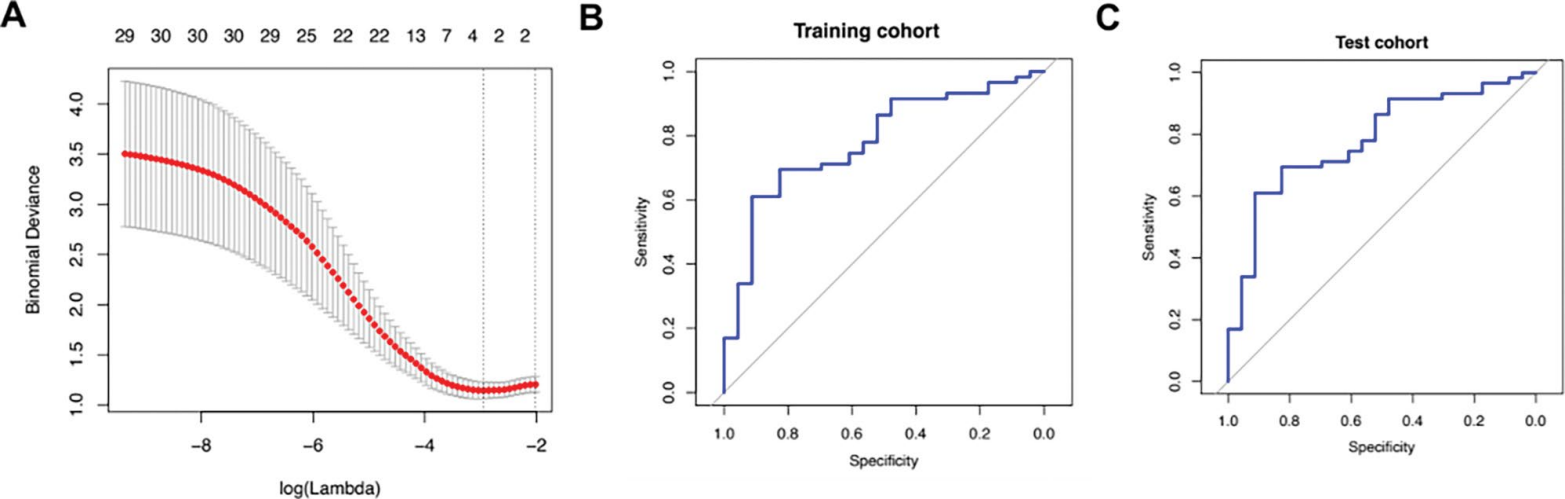
**A** Lasso regularization plot showing the log-transformed values of the regularization parameter, lambda (λ), on the X axis, which controls the penalty applied to the coefficients. The y axis shows the cross-validated error or deviance. The optimal lambda is chosen where the cross-validation error is minimized; **B and C** ROC plots illustrating specificity and sensitivity of the radiomics score established upon Lasso analysis for the distinction between high-grade and low-grade UTUC

Next, testing was carried out in the 117 patients with high-grade and low-grade UTUC to differentiate between both tumor entities. After performing the penalized regression model, a total of four features remained in the radiomic score. A sensitivity of 61% and a specificity of 91.3% was calculated for the differentiation between high-grade and low-grade UTUC for the training cohort (AUC: 0.78, Radiomics score cutoff value: 0.725). For the test cohort, a sensitivity of 57.7% and a specificity of 88.9% was computed (AUC 0.68, Radiomics score cutoff value: 0.729, see Fig. 4).

## Discussion

This study demonstrates that radiomics-based image analysis enables non-invasive differentiation of UTUC and RCC in preoperative venous-phase CT datasets with high diagnostic accuracy validated by histopathology.

The findings underscore the feasibility of radiomic feature extraction and machine learning-based analysis as a novel, non-invasive tool in the preoperative diagnostic workflow, even when applied to heterogeneous CT datasets from multiple institutions. The radiomic score achieved moderate sensitivity (81%) and specificity (80%) for differentiating UTUC from RCC, indicating that while its discriminative ability is not yet optimal, it may provide valuable diagnostic support when combined with established modalities such as endourological diagnostics. Importantly, the high specificity (88.9%) observed in differentiating high-grade from low-grade UTUC suggests that radiomics analysis could reliably exclude high-grade carcinoma, guiding clinical decisions, especially for kidney-sparing procedures. This is clinically relevant given the distinct surgical management strategies for high-grade and low-grade tumors [1].

Furthermore, our study evaluated the differentiation between both clear cell RCC (ccRCC) and high-grade UTUC, as well as non-clear cell RCC (nccRCC) and UTUC. Clear cell RCC is the most common subtype and typically exhibits strong, characteristic contrast enhancement, which often facilitates diagnosis. Thus, distinguishing ccRCC from high-grade UTUC is clinically relevant, particularly in cases where endoscopic or cytological confirmation is not immediately available. To address concerns regarding potential bias introduced by easier-to-diagnose tumors, we also performed a focused sub-analysis excluding ccRCC. Notably, the model maintained a good discriminative performance in differentiating nccRCC from UTUC, supporting its potential applicability even in diagnostically more ambiguous cases.

The relatively low sensitivity in distinguishing high-grade from low-grade UTUC could be attributed to the small number of low-grade UTUC cases included, likely due to the rarity of these tumors requiring imaging in our center. Low-grade UTUC cases are often less locally advanced and thus may not present diagnostic challenges comparable to the image-based discrimination between high-grade UTUC and RCC with renal pelvis infiltration.

Few studies have applied radiomic analysis to differentiate UTUC from RCC. Zhai et al. investigated a random forest-based radiomics model, a clinical model, and a combination of both for the differentiation of RCC and pyelocaliceal UTUC in a smaller patient cohort. In line with our results, the authors found both the radiomics model and the combined radiomics/clinical model to be powerful tools for the differentiation of UTUC and RCC (testing cohort: AUC 0.90 for the radiomics model and AUC 0.90 for the combined model, respectively). Both this and the above-mentioned studies demonstrate that differentiation of UTUC and RCC is feasible in both a Western European and an East Asian patient population. Notably, our study included three times more patients (236 vs. 80), potentially increasing the generalizability of our results. Although integration of clinical and imaging data often is of major importance, the only clinical feature proving independent in the study by Zhai et al. was painless hematuria, and combining the clinical model and the radiomics model did not improve AUC compared to radiomics alone. Consequently, the study by Zhai et al. confirms the limited value of clinical parameters in the differentiation of UTUC and RCC and therefore underlines the clinical need for non-invasive imaging diagnostic biomarkers [13].

**Fig. 5:**
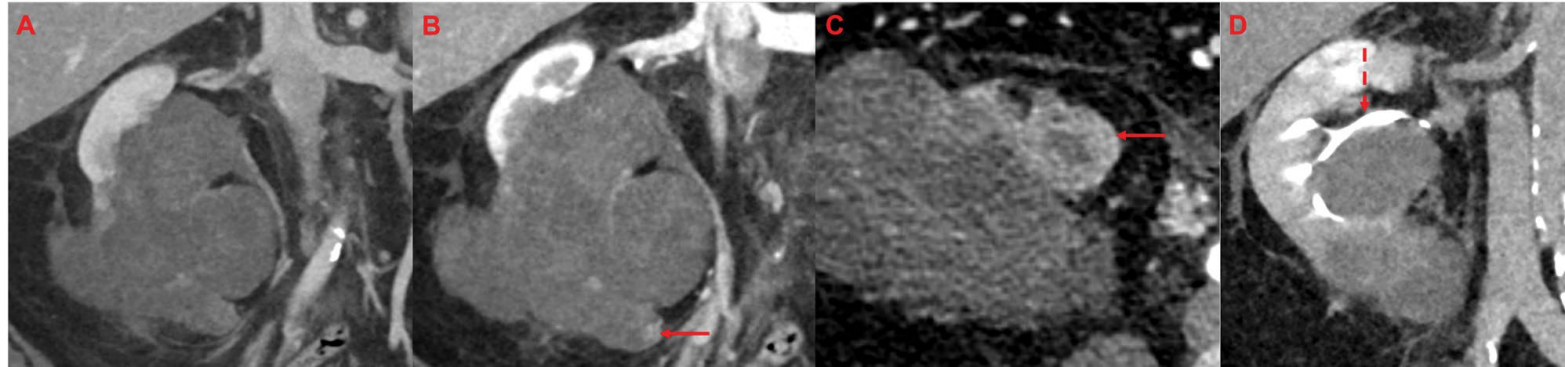
Locally advanced renal mass as a diagnostic challenge. Preoperative CT (**A** coronary venous phase, **B** coronary corticomedullary phase, **C** axial corticomedullary phase, **D** coronary urographic phase) demonstrates a large necrotic renal mass. The corticomedullary phase demonstrates only subtle arterial enhancement of the inferior part of the tumor (**B** and **C**, arrows). In the urographic phase (**D**), the renal pelvis is displaced (**D**, dashed arrow) but it remains unclear if the renal calyces are infiltrated. The patient underwent nephrectomy and postoperative histopathology revealed a locally advanced chromophobe RCC (pT3a pN0)

The aim of this study was to evaluate radiomics as an innovative, complementary approach to established diagnostic tools such as conventional imaging—particularly in cases with inconclusive or ambiguous findings, even on multiphase imaging. Figure 5 presents an example of a diagnostically ambiguous renal mass—outside of the study cohort—to illustrate the challenges that can persist even with multiphase CT imaging.

By providing a quantitative, reproducible framework for image analysis, radiomics has the potential to support more standardized diagnostic workflows and reduce interobserver variability in the assessment of UTUCs.

Radiomics may help overcome some of the limitations associated with traditional biopsy, such as sampling bias resulting from intratumoral heterogeneity, and may also be valuable in settings where cytology or endoscopic procedures are not readily available. For example, histopathological examination of nephroureterectomy specimens can reveal high-grade tumor components despite initial low-grade findings in limited biopsy samples [14]. Radiomics, as a “digital biopsy”, evaluates the entire tumor volume, offering a more comprehensive representation of tumor biology [15].

The time from diagnosis to surgical intervention is crucial especially in UTUC [16]. The use of digital biopsies could streamline the diagnostic workflow for ambiguous renal lesions. By extracting diagnostic data from preoperative imaging, radiomics could reduce the need for invasive biopsies and the associated waiting times for histopathological analysis. toward which radiomic analysis might be a first step in the development of appropriate technologies. Patients might therefore be able to proceed directly to the appropriate intervention or treatment with potential incorporation of those imaging findings into surgical workflows [17]. This expedited process could enhance patient experience by reducing anxiety and distress while improving oncological outcomes through timely intervention [18]. Additionally, minimizing reliance on invasive procedures could reduce healthcare costs, although the time and expense of radiomic image processing must be considered in compensation models for radiology services.

Several limitations of this study must be acknowledged. First, we performed a retrospective single-center analysis as proof-of-concept and therefore might have a reduced external validity of our results [19]. Prospective, multi-center studies are necessary for robust validation. While the inclusion of imaging from different institutions demonstrates algorithm robustness, variability in imaging protocols may have influenced the results.

Second, cases in which tumors were biopsied rather than fully resected may introduce bias due to the absence of whole-tumor histopathology.

Another limitation of this study is the absence of feature harmonization across imaging data, which may affect inter-institutional comparability; future studies will incorporate established harmonization techniques to address this issue.

While the primary aim of this study was to assess the ability of a radiomics model to differentiate between UTUC and RCC, we acknowledge the critical role of established clinical and radiological diagnostics. Future studies should therefore integrate preoperative clinical or radiological assessments and evaluate the added value of radiomics in confirming or refining these initial diagnoses.

While this proof-of-concept study focused on quantitative radiomic features, we acknowledge that tumor size may influence feature distributions and recognize the higher tumor volumes observed in the UTUC cohort. Future studies should account for tumor size more systematically to further validate and refine these findings.

Additionally, as noted above, the small number of low-grade UTUC cases reflects the rarity of these tumors in our cohort, potentially affecting the analysis of this subgroup. This may result in a limited utility of radiomics for guiding conservative treatment planning in UTUCs. Furthermore, it remains unclear whether necrotic areas should be excluded from segmentation, as their inclusion might impact feature extraction and model performance.

## Conclusion

This proof-of-concept study demonstrated that a radiomics-based approach utilizing machine learning for feature selection and classification can reliably differentiate between RCC and UTUC. These findings highlight the potential of radiomics as a non-invasive diagnostic tool for upper urinary tract tumors, however, the differentiation between low- and high-grade UTUCs proved more challenging. In order to fully establish this technique in clinical practice, validation through prospective multicenter trials and further optimization of the methodology are essential.

## Electronic supplementary material

Below is the link to the electronic supplementary material.

Supplementary Material 1

## Data Availability

The datasets used and analyzed during this study are available from the corresponding author on reasonable request.

## Abbreviations

AUC: Area under the curve
ccRCC: Clear cell renal cell carcinoma
CT: Computed tomography
FISH: Fluorescence in situ hybridization
ICC: Intraclass correlation coefficient
IRV: Interreader variability
LASSO: Least absolute shrinkage and selection operator
nccRCC: Non–clear cell renal cell carcinoma
RCC: Renal cell carcinoma
ROI: Region of interest
UTUC: Upper urinary tract urothelial carcinoma
