# Supplementary material for: Radiomics-based differentiation of upper urinary tract urothelial and renal cell carcinoma in preoperative computed tomography datasets
Source: BMC Med Imaging. 2025 May 30;25:196. doi: 10.1186/s12880-025-01727-9 (PMC12123764; doi:10.1186/s12880-025-01727-9)
Supplement: Supplementary file 1 — Supplementary Material 1 [file 12880_2025_1727_MOESM1_ESM.docx]

**Supplementary material**

**Supplementary Table 1:** First- and second-order radiomic features extracted from renal tumors (n=59)

| Firstorder.histogram.Coefficient.variation |
| --- |
| Firstorder.histogram.Entropy |
| Firstorder.histogram.Interquartile.range |
| Firstorder.histogram.Kurtosis |
| Firstorder.histogram.Max |
| Firstorder.histogram.Maximum.histogram.gradient |
| Firstorder.histogram.Maximum.histogram.gradient.intensity |
| Firstorder.histogram.Mean |
| Firstorder.histogram.Mean.abs.deviation |
| Firstorder.histogram.Median.abs.deviation |
| Firstorder.histogram.Min |
| Firstorder.histogram.Minimum.histogram.gradient |
| Firstorder.histogram.Minimum.histogram.gradient.intensity |
| Firstorder.histogram.P10th |
| Firstorder.histogram.P25th |
| Firstorder.histogram.P50th |
| Firstorder.histogram.P75th |
| Firstorder.histogram.P90th |
| Firstorder.histogram.Quartile.coefficient.dispersion |
| Firstorder.histogram.Range |
| Firstorder.histogram.Robust.mean.abs.deviation |
| Firstorder.histogram.Skewness |
| Firstorder.histogram.Std |
| Firstorder.histogram.Uniformity |
| Firstorder.intensity.Coefficient.variation |
| Firstorder.intensity.Interquartile.range |
| Firstorder.intensity.Kurtosis |
| Firstorder.intensity.Max |
| Firstorder.intensity.Mean.abs.deviation |
| Firstorder.intensity.Min |
| Firstorder.intensity.P10th |
| Firstorder.intensity.P25th |
| Firstorder.intensity.P50th |
| Firstorder.intensity.P75th |
| Firstorder.intensity.P90th |
| Firstorder.intensity.Quartile.coefficient.dispersion |
| Firstorder.intensity.Range |
| Firstorder.intensity.Robust.mean.abs.deviation |
| Firstorder.intensity.Root.mean.square |
| Firstorder.intensity.Skewness |
| Firstorder.intensity.Std |
| Glcm.Angular.second.moment |
| Glcm.Correlation |
| Glcm.Difference.average |
| Glcm.Difference.entropy |
| Glcm.Difference.variance |
| Glcm.Dissimilarity |
| Glcm.Information.correlation.1 |
| Glcm.Information.correlation.2 |
| Glcm.Inverse.difference |
| Glcm.Inverse.difference.moment |
| Glcm.Inverse.difference.moment.normalised |
| Glcm.Inverse.difference.normalised |
| Glcm.Inverse.variance |
| Glcm.Joint.average |
| Glcm.Joint.entropy |
| Glcm.Joint.maximum |
| Glcm.Std |
| Glcm.Sum.of.averages |

**Supplementary table 2:** Radiomic features included in the Lasso regression model after IRV analysis (n=28)

| Firstorder.histogram.Entropy |
| --- |
| Firstorder.histogram.Kurtosis |
| Firstorder.histogram.Max |
| Firstorder.histogram.Maximum.histogram.gradient |
| Firstorder.histogram.Mean |
| Firstorder.histogram.Mean.abs.deviation |
| Firstorder.histogram.Median.abs.deviation |
| Firstorder.histogram.Minimum.histogram.gradient |
| Firstorder.histogram.Range |
| Firstorder.histogram.Robust.mean.abs.deviation |
| Firstorder.histogram.Skewness |
| Firstorder.histogram.Std |
| Firstorder.intensity.Kurtosis |
| Firstorder.intensity.Max |
| Firstorder.intensity.Mean.abs.deviation |
| Firstorder.intensity.Range |
| Firstorder.intensity.Robust.mean.abs.deviation |
| Firstorder.intensity.Root.mean.square |
| Firstorder.intensity.Skewness |
| Firstorder.intensity.Std |
| Glcm.Difference.average |
| Glcm.Difference.entropy |
| Glcm.Difference.variance |
| Glcm.Dissimilarity |
| Glcm.Joint.average |
| Glcm.Joint.entropy |
| Glcm.Std |
| Glcm.Sum.of.averages |

**Supplementary Document 1**

STROBE Statement—Checklist of items that should be included in reports of ***cohort studies***

|  | Item No | Recommendation | Page No |
| --- | --- | --- | --- |
| **Title and abstract** | 1 | (*a*) Indicate the study’s design with a commonly used term in the title or the abstract |  |
|  |  | (*b*) Provide in the abstract an informative and balanced summary of what was done and what was found |  |
| Introduction | | | |
| Background/rationale | 2 | Explain the scientific background and rationale for the investigation being reported |  |
| Objectives | 3 | State specific objectives, including any prespecified hypotheses |  |
| Methods | | | |
| Study design | 4 | Present key elements of study design early in the paper |  |
| Setting | 5 | Describe the setting, locations, and relevant dates, including periods of recruitment, exposure, follow-up, and data collection |  |
| Participants | 6 | (*a*) Give the eligibility criteria, and the sources and methods of selection of participants. Describe methods of follow-up |  |
|  |  | (*b*) For matched studies, give matching criteria and number of exposed and unexposed |  |
| Variables | 7 | Clearly define all outcomes, exposures, predictors, potential confounders, and effect modifiers. Give diagnostic criteria, if applicable |  |
| Data sources/ measurement | 8* | For each variable of interest, give sources of data and details of methods of assessment (measurement). Describe comparability of assessment methods if there is more than one group |  |
| Bias | 9 | Describe any efforts to address potential sources of bias |  |
| Study size | 10 | Explain how the study size was arrived at |  |
| Quantitative variables | 11 | Explain how quantitative variables were handled in the analyses. If applicable, describe which groupings were chosen and why |  |
| Statistical methods | 12 | (*a*) Describe all statistical methods, including those used to control for confounding |  |
|  |  | (*b*) Describe any methods used to examine subgroups and interactions |  |
|  |  | (*c*) Explain how missing data were addressed |  |
|  |  | (*d*) If applicable, explain how loss to follow-up was addressed |  |
|  |  | (*e*) Describe any sensitivity analyses |  |
| Results | | |  |
| Participants | 13* | (a) Report numbers of individuals at each stage of study—eg numbers potentially eligible, examined for eligibility, confirmed eligible, included in the study, completing follow-up, and analysed |  |
|  |  | (b) Give reasons for non-participation at each stage |  |
|  |  | (c) Consider use of a flow diagram |  |
| Descriptive data | 14* | (a) Give characteristics of study participants (eg demographic, clinical, social) and information on exposures and potential confounders |  |
|  |  | (b) Indicate number of participants with missing data for each variable of interest |  |
|  |  | (c) Summarise follow-up time (eg, average and total amount) |  |
| Outcome data | 15* | Report numbers of outcome events or summary measures over time |  |
